# Chronic Pancreatitis and Neoplasia: Correlation or Coincidence

**DOI:** 10.1155/1997/89374

**Published:** 1997

**Authors:** G. N. Zografos, A. G. Bean, M. Bowles, R. C. N. Williamson

**Affiliations:** Department of Surgery Royal Postgraduate Medical School Hammersmith Hospital London UK

## Abstract

Any link between pancreatic carcinoma and chronic pancreatitis could reflect the malignant potential of a chronic inflammatory process. Four patients with ductal adenocarcinomas had a long history of pancreatic pain (median duration 5 years) and showed clearcut evidence of chronic pancreatitis “downstream” of the tumour. Four were alcoholics and two heavy smokers. These four cases arose within a surgical series of approximately 250 patients with chronic pancreatitis, giving an incidence of 1.6 per cent. The incidence and anatomical distribution of carcinoma and chronic pancreatitis could possibly be consistent with a casual relationship.


*HPB Surgery*, 1997, Vol.10, pp. 235–239
Reprints available directly from the publisher
Photocopying permitted by license only

© 1997 OPA (Overseas Publishers Association)
Amsterdam B.V. Published in The Netherlands
by Harwood Academic Publishers
Printed in India

### *Case Report*

### Chronic Pancreatitis and Neoplasia: Correlation or Coincidence

G. N. ZOGRAFOS, A. G. BEAN, M. BOWLES and R. C. N. WILLIAMSON

*Department of Surgery Royal Postgraduate Medical School Hammersmith Hospital, London, U.K.*

*(Received 13 October 1995)*

**Any link between pancreatic carcinoma and chronic pancreatitis could reflect the malignant potential of a chronic inflammatory process. Four patients with ductal adenocarcinomas had a long history of pancreatic pain (median duration 5 years) and showed clear-cut evidence of chronic pancreatitis "downstream" of the tumour. Four were alcoholics and two heavy smokers. These four cases arose within a surgical series of approximately 250 patients with chronic pancreatitis, giving an incidence of 1.6 per cent. The incidence and anatomical distribution of carcinoma and chronic pancreatitis could possibly be consistent with a casual relationship.**

*Keywords:* Chronic pancreatitis, pancreatitis cancer, pancreatic adenocarcinoma

### INTRODUCTION

Chronic pancreatitis and carcinoma of the pancreas are each relatively common. The incidence of chronic pancreatitis in Western countries is approximately 4 per 100,000; the death rate and thus the incidence for pancreatic cancer is 10 per 100,000[1]. Although chronic pancreatitis has been suggested as a risk factor for pancreatic carcinoma in several studies[2,3], this association is still unproved.

It is well known that a carcinoma in the head of pancreas can obstruct the pancreatic duct causing either an attack of acute pancreatitis or, more commonly, a type of chronic pancreatitis that may be termed "distal obstructive pancreatopathy". This condition can be difficult to differentiate from primary chronic pancreatitis on histological examination[4,5]. These obstructive changes "upstream" of the tumour can also cause diagnostic difficulties for the clinician in obtaining a representative pancreatic biopsy.

We present four patients in whom long-standing chronic pancreatic was complicated by the development of pancreatic (ductal) carcinoma. These cases may suggest a positive aetiological correlation.

### PATIENTS AND RESULTS

### Case 1

A 39 year old man was admitted for investigation of abdominal pain. He had a history of previous alcohol ingestion for 12 years and

Correspondence to: Mr. George Zografos 14 Agias Filotheis str., 152 37 Filothey Athens GREECE.

was a smoker. He had a 10-years history of recurrent severe episodes of abdominal pain. Some of these episodes were established as acute pancreatitis. He was then referred for further treatment to Hammersmith Hospital. He had exocrine insufficiency but was not diabetic. Abdominal CT scan showed several well defined pancreatic cysts with the largest cyst measuring 3 cm, in the body of pancreas. Endoscopic retrograde pancreatography showed a grossly abnormal pancreatic duct in the head and tail of the pancreas. On operation, the head of the pancreas was found to be relatively normal, but the gland distally to the neck was severely diseased with a 4–5 cm pseudocyst. A 60% distal pancreatectomy and a splenectomy were performed. Histology showed chronic inflammatory infiltrate particularly associated with a haemorragic and partly necrotic wall.

Following surgery, his pain persisted, despite coeliac plexus block and intrapleural buvicaine block. He underwent a completion pylorus-preserving proximal pancreatoduodenectomy one year after the initial operation. The residual head of pancreas was found to be indurated and inflammed. Histology showed chronic pancreatitis. To the left of the midline, away from the residual pancreatic tissue there was a plaque adherent to the stomach which was found to be poorly differentiated adenocarcinoma of pancreatic primary origin. Following surgery his disease rapidly disseminated and the patient died 3 months later.

### Case 2

A sixty-three year old lady was referred to Hammer-smith for further investigation of chronic pancreatitis. She had suffered from recurrent epigastric pain for 5 years and she had lost six stones in weight during the preceding 18 months, associated with exocrine insufficiency. She had recently become an insulin-dependent diabetic. Her pain gradually became more frequent, requiring oral pethidine. She had a previous alcohol intake and was a smoker. ERCP done 18 months prior to her referral to Hammersmith had demonstrated dilatation of the pancreatic duct consistent with chronic pancreatitis. On admission, CT scan revealed massive ascites and bilateral pleural effusions. The contours of the pancreas could not be clearly defined and the differential diagnosis was of severe acute pancreatitis or pancreatic cancer. The visceral angiography showed occlusion of the splenic artery and middle colic artery encasement. The ascites was drained and the fluid sent for cytology. The diagnosis of inoperable pancreatic cancer was then established and the patient was referred to the oncologists.

### Case 3

A 44 year-old man was referred to Hammersmith Hospital with a history of more than one year of epigastric pain accompanied by weight loss and endocrine as well as exocrine pancreatic insufficiency. He had been a heavy drinker in the past, consuming up to a bottle of brandy a day. The ERCP showed calcification in the head and tail of the pancreas with dilated main duct to the body which ended in a stricture with no obvious filling of the distal duct. Changes were thought to be consistent with chronic pancreatitis.

The abdominal CT-scan showed calcification in the tail of the pancreas and overlying inflammatory tissue posterior to the spleen. A visceral angiogram showed occlusion of the splenic vein, a normal portal vein and superior mesenteric vein, whereas the splenic artery appeared to be either encased or tethered.

At laparotomy a huge mass was found in the tail of the pancreas, invading the fundus of the stomach, the colon and the ligament of Treitz, with multiple deposits in both liver lobes. Consequently, a palliative gastrojejunostomy, an ileo-colonic anastomosis and a coeliac plexus block were performed. Histology of pancreatic nodule showed poorly differentiated adenocarcinoma.

### Case 4

A 58 years old publican who was admitted in January 1979 with a 2-years history of epigastric pain of a continuous nature, radiating to the lumbar spine. He had a long history of alcohol consumption. The diagnosis of acute pancreatitis was established, but as the symptoms continued he underwent an abdominal exploration 3 weeks later. The pancreas was found thickened throughout the gland, involving head, body and tail. Postoperatively the patient continued to suffer from acute pancreatitis and he was referred for further treatment. Pancreatic exocrine impairment was found whereas ERCP suggested a probable stricture in the head of the gland. The patient was reexplored in April 1979, with a presumptive diagnosis of chronic generalised pancreatitis. The findings were of gross generalised chronic pancreatitis with a small calibre duct. A total pancreaticoduodenectomy, partial gastrectomy and splenectomy was performed. Histology revealed a moderately well differentiated invasive pancreatic adenocarcinoma arising from the neck of the pancreas. There was fibrosis and chronic inflammation in the head, body and tail of the pancreas. The regional lymph nodes were free of tumour.

### DISCUSSION

Despite advances in pancreatic imaging, the differential diagnosis between chronic pancreatitis and pancreatic cancer can be difficult to establish. In three of the four patients with ductal carcinoma the diagnosis of malignancy was only established at operation, and in the two with resection it was an unexpected histological finding. Moossa and Co-workers reported four early cancers as incidental findings after resection for pancreatitis among 64 cases of pancreatic cancer[6]. The present series derives from approximately 250 consecutive patients with chronic pancreatitis treated by the same surgeon (RCNW) over a 12 year period. There were 4 cases of pancreatic adenocarcinoma (1.6%).

TABLE 1 Cases of chronic pancreatitis and pancreatic ductal carcinoma.

| *Case no:* | *1* | *2* | *3* | *4* |
|---|---|---|---|---|
| Sex/Age.(yrs) | M/39 | F/63 | M/44 | M/58 |
| Alcohol | yes | yes | yes | yes |
| Smoking | yes | yes | no | no |
| Duration of pain | 10yrs | 5yrs | >1yr | 2yrs |
| ERCP | Grossly Irreg. MPD | Irreg.MPD+ ectasia | Dilated MPD in head. Stricture In body | Stenosis MPD In body |
| CT Scan | Pseudocyst DP | Irresectable cancer (18 mo after ERCP) | Calcification head and tail | – |
| Operation | 1.Resection DP 2.Resection PP | None | Bypass | Total pancreatectomy |
| Location of tumour in pancreas | Body | Body | Tail | Neck |
| Distribution of chronic pancreatitis | Generalised | Generalised | Generalised | Gerneralised |
| Outcome | Died at 3 mo | Died at 1 mo | Died at 3 mo | Died at 3 mo |

DP=Distal pancreas; PP-Proximal pancreas; MPD=Main pancreatic duct

The aetiopathological correlation between chronic pancreatitis and pancreatic cancer remains uncertain. In support, the incidence of cancer varied between 1.2–6.0 per cent in six series of patients with chronic pancreatitis who were followed up for 1–10 years postoperatively[7–12]. Of the 23 patients involved, 8 probably had concomitant cancers that were unrecognised at operation because the diagnosis was established within a few months. In an international study of 1552 patients with chronic pancreatitis, 29 had evidence of cancer of the pancreas two or more years after the diagnosis of pancreatitis[13]. The expected number was 1.76, giving a standardised incidence ratio 16.5, though there is some potential for misclassification and detection bias in the figure[14]. The cumulative increase in risk was seen in patients with both alcoholic and nonalcoholic disease. In a recent study both chronic and acute pancreatitis were found to be a significant risk for development of pancreatic cancer[15]. Histopathological studies have shown a relationship between dysplasia and chronic pancreatitis, with a possible transition from dysplasia to carcinoma *in-situ* and thence to invasive carcinoma[16].

Aggressive surgical approach to chronic pancreatitis based on fine needle biopsies and CA 19.9 determinations has been advocated[17].

Not all the evidence points to an association between chronic pancreatitis and pancreatic cancer. In an early series of 64 patients no relationship was found[18]. A recent case-controlled study that specifically addressed the role of medical history in pancreatic cancer found no convincing evidence for chronic pancreatitis as a predisposing condition[19].

Two types of chronic pancreatitis that clearly predispose to carcinoma are the rare familiar form of the disease[20] and mucinous ductal ectasia[21]. The latter was associated with segmental pancreatitis and early invasive carcinoma in one of our patients, who has been excluded from the study. In all patients with ductal carcinoma, an aetiological role for chronic pancreatitis is postulated by the following data:

1. a long history of pancreatic pain (from 1 to 10 years).
2. a clear-cut aetiological agent, namely alcoholism.
3. evidence of generalised chronic pancreatitis, i.e. inflammatory disease "downstream" of the tumours which were situated at or to the left of the midline. Other possible aetiological factors were cigarette smoking (in two of the four) and perhaps alcoholism itself, although the association is weak[19]. Either some forms of pancreatitis are a precursor to pancreatic cancer of shared risk factors for both diseases (eg., cigarette smoking) may also be involved[22].

### *References*

[1] Di Magno, E.P. (1988). Early diagnosis of chronic pancreatitis and pancreatic cancer. *Med. Clin. N. Am.*, **72(S)**, 979–992.

[2] Mikal, S. and Cambell A.J.A. (1950). Carcinoma of the pancreas. *Surgery*, **28**, 963.

[3] Moldow, E.R. and Connelly, R.P. (1968). Epidemiology of pancreatic cancer in Connecticut. *Gastroenterology.*, **55**, 677–682.

[4] Fontham, E.T. and Correa, R.P. (1989). Epidomiology of pancreatic cancer. *Surg. Clin N. Am.*, **69(3)**, 551–567.

[5] Lowes, J.R., Rode, J., Lees, W.R., Russe, R.C.G. and Cotton, P.B. (1988). Obstructive pancreatitis. *Br. J. Surg.*, **75**, 1129–1133.

[6] Moossa, A.R. and Levin, B. (1981). The diagnosis of early pancreatic cancer. *Cancer*, **47**, 1688–1697.

[7] Amman, R.W., Akovbiants, A., Largiader, F. and Shueler, G. (1984). Course and outcome of chronic pancreatitis. Longitudinal study of a mixed medical-surgical series of 245 patients. *Gastroenterology*, **86**, 820–828.

[8] Greenlee, H.B., Prinz, R.A. and Aranha, G.V. (1990). Long-term results of side-to-side pancreaticojejunostomy. *World. J. Surg.*, **14**, 70–76.

[9] Bradley, E.L. (1987). Long-term results of pancreatojejunostomy in patients with chronic pancreatitis. *Am. J. Surg.*, **153**, 207–217.

[10] Moreaux, J. (1984). Long-term follow-up study of 50 patients with pancreaticoduodenectomy for chronic pancreatitis. *World. J. Surg.*, **8**, 3446–353.

[11] Prins, R.A. and Greenlee, H.B. (1981). Pancreatic duct drainage in 100 patients with chronic pancreatitis. *Ann. Surg.*, **194**, 313–320.

[12] Taylor, R.H., Bagley, F.H., Braasch, J.W. and Warren, K.W. (1981). Ductal drainage or resection for chronic pancreatitis. *Am. J. Surg.*, **141**, 28–33.

[13] Lowenfels, A.B., Maisonneuve, P. and Cavallini, G.

(1993). Pancreatitits and the risk of pancreatic cancer. *N. Engl. J. Med.*, **328**, 1485–1486.
[14] Gold, E.B. (1993). JL Chronic pancreatitis and pancreatic cancer. *N. Engl. J. Med.*, **328**, 1485–1486.
[15] Bansal, P., and Sommenbery, A. (1995). Pancretitis is a risk factor for pancreatic cancer. *Gastroenterology*, **109**, 247–251.
[16] Cylwik, B., Nowak, H.Y., Puchalski, Z. and Barcky, K.J. (1985). Chronic pancreatitis as a predisposing factor in the development of pancreatic cancer. Histological and histochemical studies. *Zentralbl. Allg. Pathol.*, **130**, 217–214.
[17] Haas, O., Guiliard, G., Rat, P., Friedman, S. and Favre, J.P. (1990). Pancreatic carcinoma developing in chronic pancreatitis: A report of four cases. *Hepatogastroenterol*, **37**, 350–351.
[18] Johnson, J.R. and Zintel, H.A. (1963). Pancreatic calcification and cancer of the pancreas. *Surg. Gynec. Obstet.*, **11**, 585–588.
[19] Gold, A.B., Gordis, A.L. and Diener, M.D., *et al.* (1985). Diet and other risk factors for pancreatitis. *Cancer*, **55**, 460–466.
[20] Warshaw, A.L. and Fernandes-Del Kastillo. (1992). Pancreatic carcinoma. *N. Engl. J. Med.*, **326**, 455–465.
[21] Bastid, C., Bernard, J.P., Sarles, H., Payan, M.J. and Sahel, J. (1991). Mucinous ductal ectasia of the pancreas: A premalignant disease and a cause of obstructive pancreatitis. *Pancreas*, **6**, 15–22.
[22] Ekbom, A., McLaughlin, J.K., Karlsson, B.M., Nyren, O., Gridley, G., Adami, H.O. and Fraumeni, J.F. (1994). Pancreatitis and pancreatic cancer: a population-based study. *J. Natl. Cancer Inst.*, **86(8)**, 625–627.

### COMMENTARY

Although the issue remains controversial, several publications including a large cohort study of 1522 patients, have suggested an increased risk of pancreatic cancer in patients with antecedent chronic pancreatitis. Any surgeon with experience in pancreatic surgery, will remember one or two cases in which an unsuspected carcinoma was found during a procedure for chronic pancreatitis. The authors of this paper present four patients in which cancer arose in a pancreas with downstream of the tumor, evidence of chronic pancreatitis, regenerating our awareness of the association between cancer and chronic pancreatitis. However, not all chronic pancreatitis develops into cancer even not in the very long-standing cases, and the majority of patients presenting with pancreatic carcinoma have no history of chronic pancreatitis. This leaves us with a tremendous diagnostic problem, since clinical presentations and radiological images in both conditions can be similar. Even histopathological differentiation of inflammatory lesions in the pancreas and cancer is complex because it can be extremely difficult to discriminate between ductal neoplasia and regenerative epithelium particularly in well-differentiated adenocarcinoma. And, even when we might dispose of a histological marker that can identify cancer with high accuracy there still remains the problem of the sampling error. How can we be sure that we are biopsying the tumor amidst an inflammatory mass? Thus, it is not very likely that we will ever find our way out of this dilemma. Until we can come up with new solutions a more aggressive approach towards resection of any suspicious lesion in chronic pancreatitis seems justified. And when the lesion proves to be benign, resection after all, is not a bad treatment for chronic pancreatitis.

Dr. T.M. Von Gulik
Acalemisch Zeikenhuis
Universteit von Amsterdam
Amsterdam
Netherlands

